# Introduction of Small Stitch Small Bite technique: a retrospective long-term follow-up

**DOI:** 10.1007/s00423-022-02530-8

**Published:** 2022-05-17

**Authors:** Harald Söderbäck, Arslan Masood, Jonas Leo, Gabriel Sandblom

**Affiliations:** 1 Department of Clinical Science and Education, Karolinska Institute, Södersjukhuset, Stockholm, Sweden; 2 Capio St Göran’s Sjukhus, kirurgexpeditionen St Görans plan 1, 11281 Stockholm, Sweden; 3 Department of Surgery, Capio St Göran’s Hospital, Stockholm, Sweden

**Keywords:** Incisional hernia, Wound dehiscence, Midline incision, Surgical technique

## Abstract

**Purpose:**

Standardization of abdominal wall closure is suggested to improve quality and reduce the risk for late abdominal wall complications. The purpose of this study was to explore the impact of a structured introduction of guidelines for abdominal wall closure on the rates of incisional hernia and wound dehiscence.

**Methods:**

All procedures performed via a midline incision in 2010–2011 and 2016–2017 at Capio St Göran’s Hospital were identified and assessed for complications and risk factors.

**Results:**

Six hundred two procedures were registered in 2010–2011, and 518 in 2016–2017. Four years after the implementation of new guidelines, 93% of procedures were performed using the standardized technique. There was no significant difference in the incidence of incisional hernia or wound dehiscence between the groups. In multivariate Cox proportional hazard analysis, BMI > 25, wound dehiscence, and postoperative wound infection were found to be independent risk factors for incisional hernia (all *p* < 0.05). In multivariate logistic regression analysis, male gender and chronic obstructive pulmonary disease were risk factors for wound dehiscence (both *p* < 0.05).

**Conclusions:**

The present study failed to show a significant improvement in rates of incisional hernia and wound dehiscence after the introduction of Small Stitch Small Bites. When introducing a new standardized technique for closing the abdomen, education and structural implementation of guidelines may have an impact in the long run. The risk factors identified should be taken into consideration when closing a midline incision to identify patients with high risk.

**Supplementary Information:**

The online version contains supplementary material available at 10.1007/s00423-022-02530-8.

## Introduction

The midline abdominal incision is widely used in abdominal surgery since it enables access to the whole abdominal cavity, spares nerves, and vessels [1, 2], and is an efficient way to open and close the abdomen [3–5]. The midline incision, however, entails a higher rate of complications compared to other abdominal incisions [3, 4, 6–8]. It is well-known that the surgical technique used for closing midline incisions affects the risk for wound complications. In 2011, Milbourn et al. presented a study favouring a technique named the Small Stitch Small Bite (SSSB) technique that dramatically decreased the incidence of incisional hernia [9]. No risk factor for incisional hernia besides the surgical technique used was found. These results were later corroborated by Deerenberg et al. [10]. SSSB is the technique recommended by the European Hernia Society for closing midline abdominal incisions since 2014 [11]. However, even when using SSSB, there is a small risk for incisional hernia and wound dehiscence. To identify patients that require more extensive measures than SSSB alone to prevent wound dehiscence and incisional hernia, risk factors for these complications must be explored.

In this study, we intended to investigate how the introduction of SSSB in 2012 as standard surgical technique influenced the rate of midline incision complications in a real-life material. We chose to include acute surgery in the study as our hospital has a profile of acute surgery. All cases performed in 2016–2017 were included in the study, to compare complications, and risk factors with cases performed prior to introduction in 2010–2011 were chosen as controls. The primary aim was to investigate the difference in incisional hernia and wound dehiscence rates between the cohorts. A secondary aim was to investigate any significant risk factors for incisional hernia and wound dehiscence.

## Methods

Based on the evidence presented by Milbourn et al. [9], Capio St Göran’s Hospital implemented SSSB as the standard technique for midline incision closure. Before the new guidelines were introduced, closing the abdominal wall was done at the surgeon’s choice. The predominating technique for closing the abdomen was a running polydioxanone (PDS) 0 loop suture, using a large needle, no notation of layers included or suture length to wound length quote. In the local guidelines (Attachment 1) revised in June 2012, the full SSSB technique is described step by step. The surgeon is instructed to use a 2–0 PDS suture on a small needle, to take small bites of only aponeurosis, to measure wound length and suture length, and to analyze the suture length to wound length ratio. The suture length, wound length, and ratio are noted in the medical record in a separate notation. To obtain a deep organizational learning of the new technique, we used a double loop learning process [12]. Before the implementation, all surgeons were educated in the new technique and got the chance to present their thoughts through local seminars where the technique was discussed. There were guest lectures by experts of the SSSB technique, and organized self-studies. During the first 6 months, the surgical technique used was monitored closely so that all surgeons cohered.

**Fig. 1:**
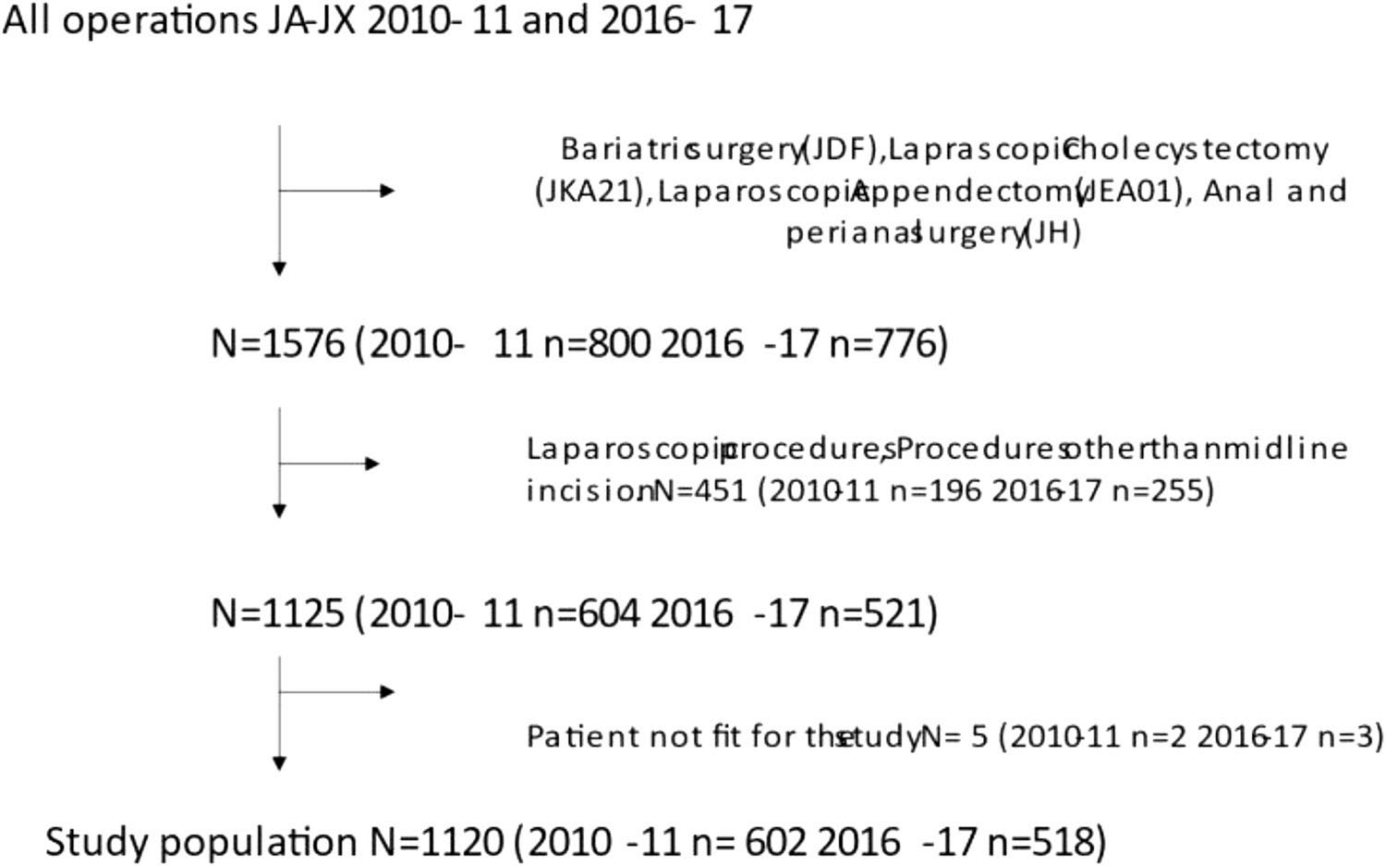
Flowchart of the study assembly

In this follow-up, all abdominal procedures performed in 2010–2011 and 2016–2017 were identified through a search for ICD10 codes in the Cambio COSMIC medical records database [13]. Assuming a difference of 5% of the prevalence of incisional hernia between the groups, the power calculation predicted that 400 patients had to be included in each group. The time frames for the study were chosen to include sufficient number of patients. Laparoscopic cholecystectomy, laparoscopic appendectomy, anal and perianal surgery, and bariatric surgeries were excluded from the search. The list was then cleared of all remaining laparoscopic surgeries and surgeries not performed through a midline incision. In a last step, patients not fitting the study, for example, where the abdomen was left open, were excluded. Figure 1 shows a flowchart of the study assembly. All remaining cases were then reviewed by two examiners. In the review, each patient record was scanned, starting at the index operation where we extracted data on potential patient-related risk factors and verified that the procedure was performed through a midline incision. The surgery records were reviewed to determine whether the study criteria were completed. A case was considered to fulfil the criteria of SSSB if an appropriate suture was used and the suture length, wound length, and ratio over 4:1 were correctly noted in the operation records. All patient records were reviewed until the end of the study period to find postoperative complications, and date of death. The endpoint “incisional hernia” was defined as either a clinically evident hernia noted on routine follow-up radiology or visits, incisional hernia accidently diagnosed clinically or by radiology on other visits to the hospital where the abdomen was examined, or surgery for incisional hernia during the follow-up period. There was no standardized protocol for follow-up for all laparotomies. Patients have been followed according to the protocols for each disease. The present follow-up of patients includes medical records and radiology reports from all contacts to the hospital until 31 March 2020. “Wound dehiscence” was defined as a clinically evident fascial dehiscence noted and treated conservatively in the postoperative period, or an acute reoperation for wound dehiscence. End of follow-up for patients in the study was defined as date of death registered in Cambio COSMIC software, 31 December the year the patient died in cases where the exact date of death was not known, or 31 March 2020. Follow-up time was set to a maximum of 3 years for all patients to make the groups more conform. Events occurring after 3 years were censored.

Statistical analyses were performed using SPSS 26.0. Analyses were performed to estimate incisional hernia and wound dehiscence rates and to assess the impact of potential risk factors. In the intention to treat analysis, patients undergoing surgery in 2016–2017 were compared to controls undergoing surgery in 2010–2011. We also performed per protocol analysis where all patients sutured with appropriate SSSB technique, with the ratio noted in the operation records, were compared to controls sutured at the surgeon’s choice. Risk factors for wound dehiscence were analyzed in uni- and multivariate logistic regression analysis; variables assumed to be risk factors at the beginning of the study were included in the multivariate analysis. Risk factors for incisional hernia were analyzed in uni- and multivariate Cox proportional hazard analysis [14]; adjustment was made for all covariates assumed to increase the risk for development of incisional hernia. Subgroup analyses were performed to investigate risk factors in each group. And potential risk factors were also analyzed for the two groups combined. All analyses were performed with an intention to treat approach comparing the early and late cohort.

## Results

Altogether, 1120 midline laparotomies were included in the study, 518 in the 2016–2017 study cohort and 602 in the 2010–2011 control cohort. Mean follow-up time was 32 and 73 months in the study and control groups, respectively. The rate of emergency surgery was approximately 60% in both groups. In the study cohort, 481 (93%) appropriate SSSB suturing was applied and a sufficient suture length to wound length ratio (≥ 4:1) was noted in the procedure notes compared to 7 (1%) in the control cohort. Altogether, 31 procedures in the study cohort and 593 procedures in the control cohort were closed at the surgeon’s choice. No significant differences in wound dehiscence rates and incisional hernia rates were seen between the two cohorts. In a per protocol analysis, cases sutured with SSSB were compared to procedures closed according to surgeon’s choice. There was no significant differences in incisional hernia or wound dehiscence between the groups in the per protocol analysis.

A total of 51 patients developed wound dehiscence, 17 (3.5%) in the SSSB group and 33 (5.3%) of surgeon’s choice closure group (*p* = 0.15). Of these, 44 required emergency reoperation, 15 in the SSSB group and 29 in the surgeon’s choice group. Nine patients (18%) with a documented wound dehiscence later developed an incisional hernia.

**Fig. 2:**
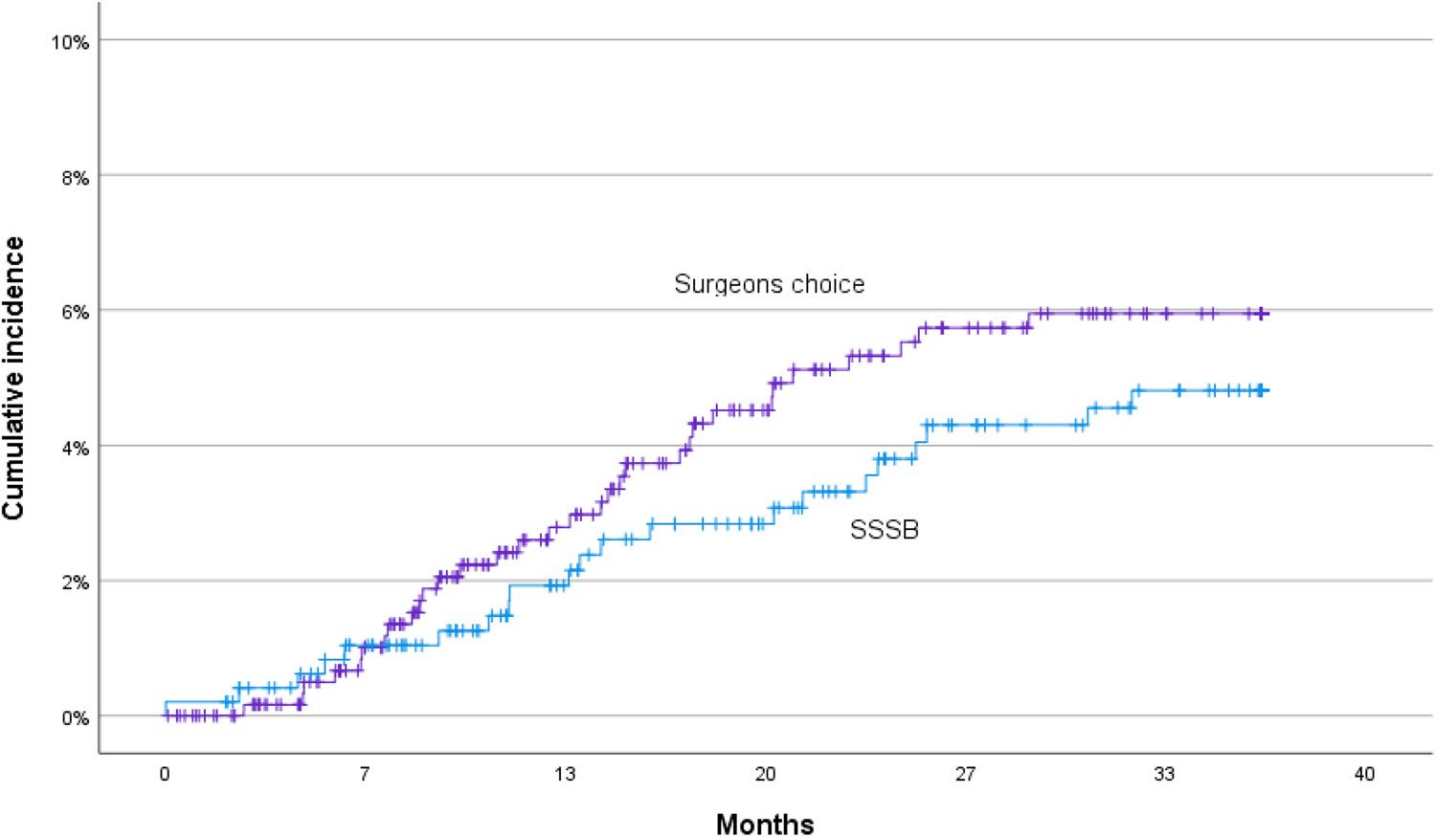
Cumulative incidence of incisional hernia related to surgical technique

In the SSSB group, 21 (4.3%) and, in the surgeon’s choice group, 32 (5.1%) developed incisional hernias (*p* = 0.52). There was no significant difference in survival analysis between the groups. Figure 2 shows Kaplan–Meier curves for the incidence of incisional hernia between the groups (*p* = 0.40 log rank). Surgical site infection (SSI) that required antibiotic treatment was seen in 15 patients (3.1%) in the SSSB group and 23 (3.7%) in the surgeon’s choice group. This difference was not statistically significant.

Table 1 shows background data and a comparison of the two study cohorts. In the subgroup multivariate analysis of the SSSB cohort, male gender (*p* = 0.03) and SSI (*p* = 0.03) were significant risk factors for wound dehiscence. BMI > 25 (*p* = 0.002), SSI (*p* < 0.001), and wound dehiscence (*p* = 0.009) proved to be risk factors for incisional hernia. Acute surgery, high age, chronic obstructive pulmonary disease (COPD), and previous midline incision did not show significant association in this group.

**Table 1:** Background data and a comparison of the two study cohorts

Background data		Closure method				Total	Sig
		Surgeons choice		SSSB			
		*N*	%	*N*	%	%	*p*
Sex	Female	325	52%	266	55%	53%	0.44
	Male	298	48%	222	45%	47%	
Age	< 70	276	44%	223	46%	45%	0.63
	≥ 70	348	56%	265	54%	55%	
ASA	1	68	14%	50	32%	12%	0.36
	2	198	40%	173	38%	39%	
	3	193	39%	193	42%	41%	
	4	32	6.5%	41	9.0%	7.7%	
	5	1	0.2%	1	0.2%	0.2%	
BMI	< 25	287	46%	250	51%	48%	0.08
	≥ 25	337	54%	238	49%	52%	
Acute surgery	Acute	372	60%	289	59%	59%	0.89
	Elective	252	40%	199	41%	41%	
Serum albumin	< 30	71	11%	64	13%	12%	0.37
Diabetes		70	11%	56	11%	11%	0.91
COPD		62	9.9%	47	9.6%	9.8%	
Previous midline incision		175	28%	128	26%	27%	0.45
Postop wound infection	Ja	23	3.7%	15	3.1%	3.4%	0.56
Type of surgery	Appendectomy	20	3.2%	17	3.5%	3.3%	0.07
	Bile ducts, liver	5	0.8%	0		0.4%	
	Explorative laparotomy	102	16%	72	15%	16%	
	Hernia	3	0.5%	1	0.2%	0.4%	
	Miscellaneous	9	1.4%	2	0.4%	1%	
	Rectal cancer	53	8.5%	18	3.7%	6.4%	
	Small intestine and colon	408	65%	361	74%	69%	
	Splenectomy	4	0.6%	2	0.4%	0.5%	
	Stomach and duodenum	20	3.2%	15	3.1%	3.1%	

Tables 2 and 3 present the results of univariable and multivariable analyses of potential risk factors for incisional hernia and wound dehiscence in the two cohorts combined.

**Table 2:** Wound dehiscence risk. Univariate and multivariate analysis

	Univariate analysis	*p*	Multivariate analysis	*p*
	Odds ratio (95% confidence interval)		Odds ratio (95% confidence interval)	
Primary procedure 2016–2017 (reference category 2010–2011)	0.70 (0.39–1.26)	0.231	0.72 (0.40–1.31)	0.283
Male (ref women)	1.94 (1.08–3.47)	0.027	2.57 (1.39–4.76)	0.003
Age ≥ 70 years (ref age < 70 years)	1.78 (0.97–3.26)	0.063	1.02 (0.99–1.03)	0.130
Acute surgery (ref elective surgery)	1.21 (0.67–2.18)	0.526	1.30 (0.71–2.38)	0.399
BMI ≥ 25 (ref BMI < 25)	0.78 (0.44–1.37)	0.385	0.76 (0.43–1.36)	0.361
COPD	2.36 (1.15–4.87)	0.020	2.40 (1.13–5.10)	0.022
Previous midline incision	1.82 (1.02–3.26)	0.043	1.82 (0.999–3.32)	0.051
Postoperative wound infection	2.56 (0.87–7.51)	0.087	2.98 (0.988–8.96)	0.053

**Table 3:** Incisional hernia risk. Univariate and multivariate analysis

	Univariate analysis	*p*	Multivariate analysis	*p*
	Hazard ratio (95% confidence interval)		Hazard ratio (95% confidence interval)	
SSSB (ref surgeons choice)	0.790 (0.455–1.370)	0.401	0.782 (0.442–1.424)	0.432
Male (ref women)	1.58 (0.92–2.72)	0.100	1.120 (0.583–2.15)	0.734
Age ≥ 70 years (ref age < 70 years)	0.79 (0.46–1.36)	0.391	0.994 (0.530–1.862)	0.984
Acute surgery (ref elective surgery)	0.57 (0.33–0.98)	0.041	0.601 (0.322–1.123)	0.984
BMI ≥ 25 (ref BMI < 25)	2.38 (1.31–4.33)	0.004	2.404 (1.315–4.394)	0.004
COPD	0.84 (0.30–2.32)	0.731	0.836 (0.293–2.38)	0.737
Previous midline incision	1.49 (0.85–2.61)	0.165	1.420 (0.799–2.53)	0.232
Postoperative wound infection	3.61 (1.54–8.46)	0.003	3.463 (1.46–8.23)	0.01
Wound dehiscence	4.49 (2.68–11.26)	0.000	5.99 (2.82–12.75)	0.000

Male gender (*p* = 0.003) and COPD (*p* = 0.022) were significant risk factors for wound dehiscence. History of a previous midline incision (*p* = 0.051) and SSI (*p* = 0.053) showed tendencies to increased risk of wound dehiscence, although not significant at the *p* < 0.05 limit.

**Fig. 3:**
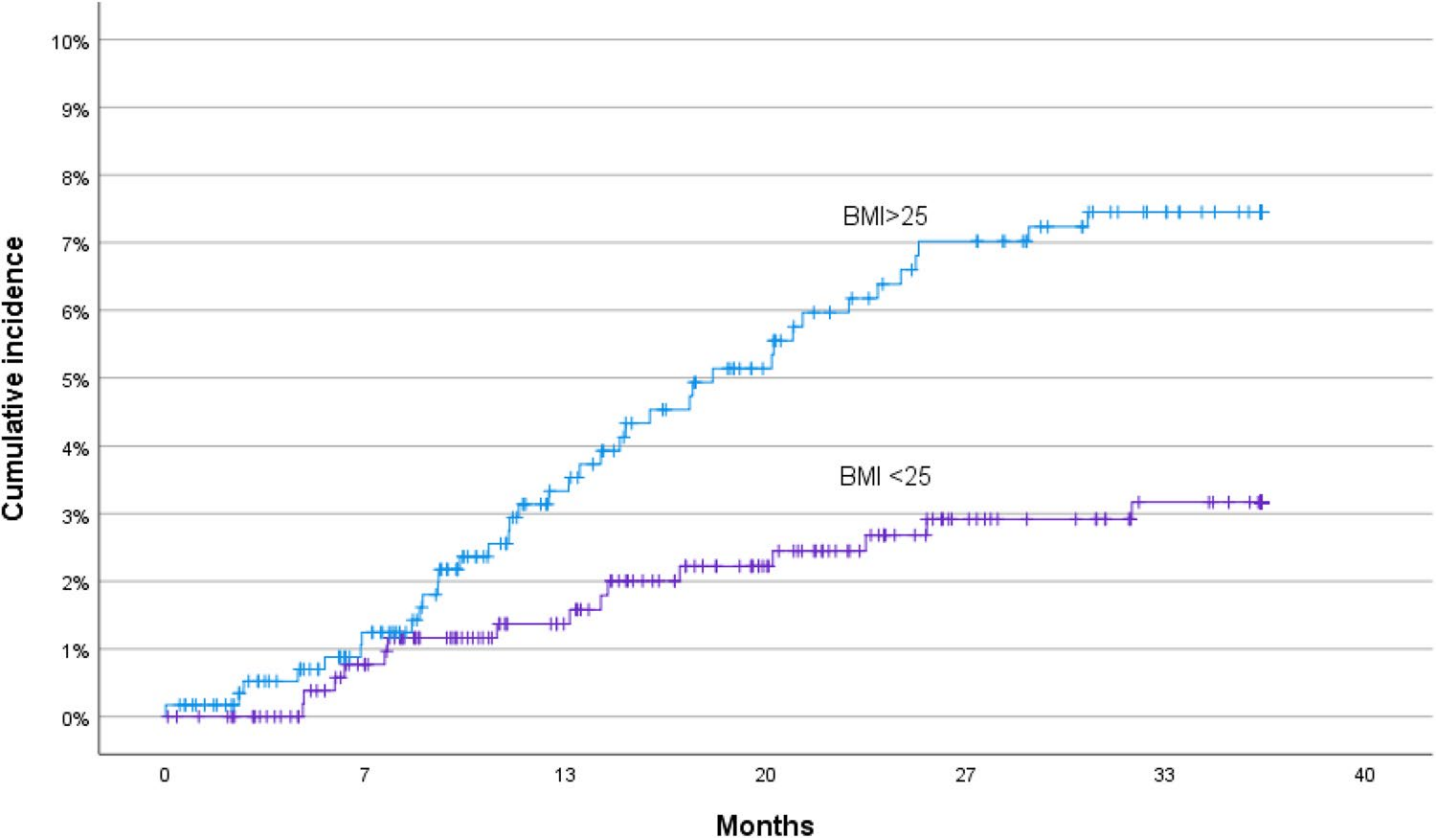
Cumulative incidence of incisional hernia in different BMI categories

**Fig. 4:**
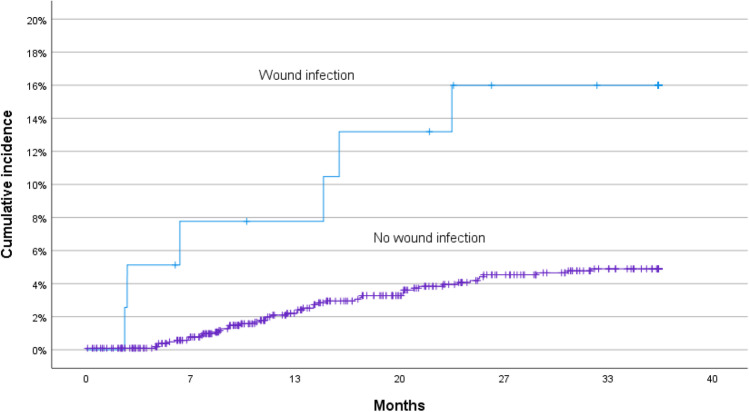
Cumulative incidence of incisional hernia related to wound infection

**Fig. 5:**
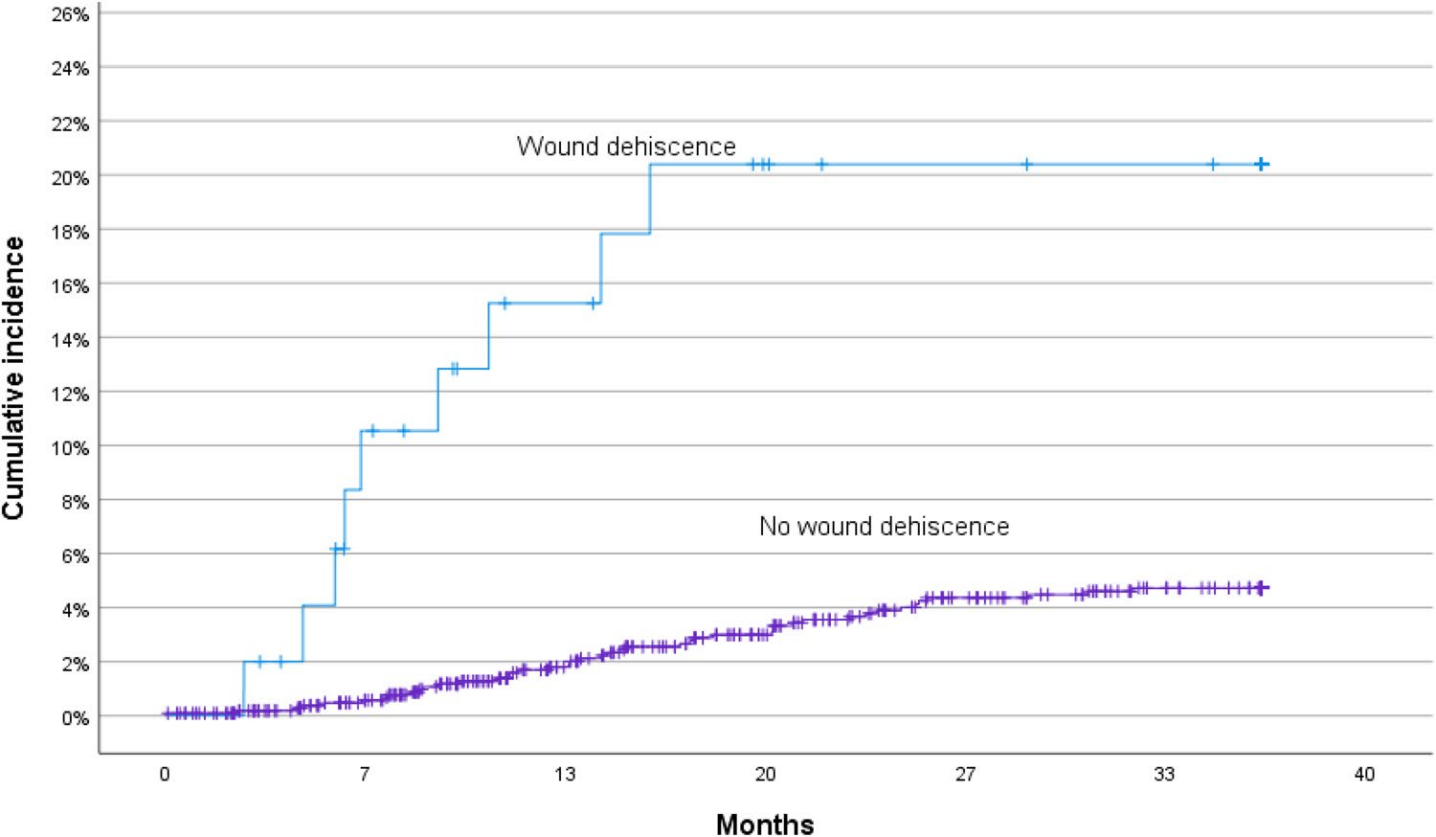
Cumulative incidence of incisional hernia related to wound dehiscence

Cox proportional hazard regression analysis identified BMI > 25 (*p* = 0.004), SSI (*p* = 0.01), and wound dehiscence (*p* < 0.001) as independent risk factors for incisional hernia. Figures 3, 4, and 5 show Kaplan–Meier curves for incisional hernia related to the significant risk factors.

## Discussion

In the present study, no significant differences in incisional hernia, wound dehiscence, or surgical site infection rates before and after the introduction of SSSB for abdominal closure were found.

Of the patients included, 60% underwent acute surgery. This may have affected the outcome as the SSSB technique is yet to be evaluated for emergency surgery. Tolstrup et al. [15] showed that there was a significant decrease in wound dehiscence rates after implementation of the SSSB in emergency surgery. This was not confirmed by this study. There is, however, still a need for more data on how the SSSB affects complications after emergency surgery.

The suegeon’s choice cohort had a low incisional hernia rate compared to literature. Since this study is retrospective, only incisional hernias that were clinically evident on follow-up visits or CT scans were registered. It is well-known that about 50% of insicional hernias are asymptomatic and only found on focused examination or radiology [10, 16, 17]. Karlsson et al. report an incisional hernia incidence of 25% after colorectal cancer surgery in a study using postoperative CT scans for the diagnosis [18]. In their study, 12% of patients needed incisional hernia surgery. By extrapolating this ratio to our study, the true incisional hernia rate in our cohorts could be expected to be at least 10%, a level that is generally considered acceptable in terms of patient safety. In the prospective studies on the SSSB [9, 10], SSSB was compared to a strict large bite technique. Since the findings of Millbourn et al. [19] were well-known in 2010, the technique diffused into clinical routine. The low rate of incisional hernia in the surgeons choice group could be due to the practice of updated techniques already prior to the introduction of SSSB as standard in 2012.

The present study failed to show a significant reduction of wound dehiscence following the introduction of SSSB. Although wound dehiscence was not an endpoint in previous studies on abdominal wall closure [9, 10], the rate was low (1% of midline laparotomies). In a recently published similar study, Albertsmeier et al. report an incident of wound dehiscence of 3.1% (1.40% in the small bite group and 4.76% in the large bite group) [20].

In the study by Milbourn, emergency as well as elective surgery was included [9]; in the studies by Deerenberg and Albertsmeier, only planned procedures were included [10, 20]. In a retrospective study, Walming et al. report a wound dehiscence rate of < 4% (3.3% in the study group and 4% in the control group) after midline laparotomy in a Swedish population that included emergency as well as planned surgery [21]. In a study on emergency laparotomies, Tolstrup et al. reduced the wound dehiscence rate from 6.6 to 3.8% after the implementation of SSSB as a standard technique [15]. The present study reports a 3.7% rate of wound dehiscence in a cohort with 60% acute surgery and where 30% of patients were previously operated through a midline incision. This finding is coherent with the findings of Walming and Tolstrup [15, 21]. Millbourn reports a significantly lower rate of wound dehiscence. In their cohort, patients with previous midline incision were excluded. As was noted by de Baux [22], comparing results is difficult due to the these differences in inclusion and exclusion criteria that affect the results.

In this study, surgical site infection rate was very low compared to the study by de Vries [23]. Albertsmeier on the other hand reports an incidence of 3.26% SSI in the small bite group [20] witch is more congruent with the present study. These differences might be due to diverging criteria for surgical site infections. In this retrospective review, only SSI that required specific antibiotic treatment was registered. There may have been several cases of SSI in patients that were already on antibiotic treatment that were not registered, which may thus have biased the outcome.

Male gender, BMI > 25, and surgical site infection proved to be significant risk factors for complication after midline incision even when the SSSB was used.

When combining the groups, male gender, COPD, previous midline incision, and postoperative wound infection were risk factors for wound dehiscence, while high BMI, postoperative wound infection, and wound dehiscence were risk factors for incisional hernia. These findings are in accordance with findings from population-based studies on patients undergoing surgery for colorectal cancer [24, 25]. Awareness of these risk factors when closing the abdomen is an important step in the prevention of midline incision complications.

There are some limitations to this study. We could not retrieve data on all relevant risk factors, e.g. information about tobacco use. There was no standardized follow-up protocol and many patients only occasionally visited the hospital in the follow-up period. Although all patients have been followed for the whole study period, there is risk for loss to follow-up as patients may have migrated, or been diagnosed with an incisional hernia at other units. However, these effects should not differ between the two groups and cannot explain why there was no difference between groups. At Capio St Göran’s hospital, there was a great focus on the SSSB in 2012. This could have led to a Hawthorne effect, i.e. of over diagnosing incisional hernia during this year, and an overestimation of the difference in incidence of incisional hernia between the surgeon’s choice and SSSB groups.

There is, as is pointed out by Garcia-Urea et al. [26], a lack of intuitive understanding of the association between meticulous care when closing the abdomen and the risk for late incisional hernia. In the present study, SSSB was applied in 93% of the procedures. In comparison, Tolstrup et al. reported 73% [15]. Bluesmen et al. conducted a questionnaire study in 2019 where Dutch surgeons were asked about the technique they used for closing midline incisions, and found that very few followed the latest guidelines [27]. This suggests that structural implementation and education using the principles of organizational learning are important and may have a long-lasting effect.

## Conclusion

The present study failed to show a significant improvement in rates of incisional hernia and wound dehiscence after the introduction of SSSB.

Results show that 4 years after the structured introduction, there was still a very good compliance to the guidelines on surgical technique.

Incidence of wound dehiscence after midline incision in a mixed population of acute and elective surgery is approximately 3.7%, and the incidence of incisional hernia in the same population is approximately 4.3%.

The present study showed that Male gender and BMI > 25 are independent risk factors for complications after midline incision, even when an SSSB technique is used. Taking these risk factors in consideration may help to identify high-risk patients where further prophylactics are indicated.

## Supplementary Information

Below is the link to the electronic supplementary material.

Supplementary file1 (DOCX 82 KB)

## Data Availability

Due to patient confidentiality, data cannot be shared.

## Code availability

SPSS code can be shared upon request.

## References

[1] Ellis H (2007). Applied anatomy of abdominal incisions. Br J Hosp Med.

[2] Luijendijk RW, Jeekel J, Storm RK, Schutte PJ, Hop WC, Drogendijk AC, Huikeshoven FJ (1997). The low transverse Pfannenstiel incision and the prevalence of incisional hernia and nerve entrapment. Ann Surg.

[3] Guillou PJ, Hall TJ, Donaldson DR, Broughton AC, Brennan TG (1980). Vertical abdominal incisions–a choice?. Br J Surg.

[4] Cox PJ, Ausobsky JR, Ellis H, Pollock AV (1986). Towards no incisional hernias: lateral paramedian versus midline incisions. J R Soc Med.

[5] Burger JWA, van ’t Riet M, Jeekel J (2002). Abdominal incisions: techniques and postoperative complications. Scand J Surg SJS Off Organ Finn Surg Soc Scand Surg Soc.

[6] Slater NJ, Bleichrodt RP, van Goor H (2012). Wound dehiscence and incisional hernia. Surg Oxf.

[7] Le Huu NR, Mege D, Ouaïssi M, Sielezneff I, Sastre B (2012). Incidence and prevention of ventral incisional hernia. J Visc Surg.

[8] Lee L, Mata J, Droeser RA, Kaneva P, Liberman S, Charlebois P, Stein B, Fried GM, Feldman LS (2018). Incisional hernia after midline versus transverse specimen extraction incision: a randomized trial in patients undergoing laparoscopic colectomy. Ann Surg.

[9] Millbourn D, Cengiz Y, Israelsson LA (2011). Risk factors for wound complications in midline abdominal incisions related to the size of stitches. Hernia J Hernias Abdom Wall Surg.

[10] Deerenberg EB, Harlaar JJ, Steyerberg EW, Lont HE, van Doorn HC, Heisterkamp J, Wijnhoven BP, Schouten WR, Cense HA, Stockmann HB, Berends FJ, Dijkhuizen FPH, Dwarkasing RS, Jairam AP, van Ramshorst GH, Kleinrensink G-J, Jeekel J, Lange JF (2015). Small bites versus large bites for closure of abdominal midline incisions (STITCH): a double-blind, multicentre, randomised controlled trial. Lancet Lond Engl.

[11] Muysoms FE, Antoniou SA, Bury K, Campanelli G, Conze J, Cuccurullo D, de Beaux AC, Deerenberg EB, East B, Fortelny RH, Gillion J-F, Henriksen NA, Israelsson L, Jairam A, Jänes A, Jeekel J, López-Cano M, Miserez M, Morales-Conde S, Sanders DL, Simons MP, Śmietański M, Venclauskas L, Berrevoet F, European Hernia Society (2015). European Hernia Society guidelines on the closure of abdominal wall incisions. Hernia J Hernias Abdom Wall Surg.

[12] Argyris C, Schön DA (1978) Organizational learning: a theory of action perspective. Addison-Wesley Publishing Company

[13] Cambio COSMIC journalsystem. https://www.cambio.se/vi-erbjuder/cosmic/. Accessed 17 Sep 2020

[14] Cox DR (1972). Regression models and life-tables. J R Stat Soc Ser B Methodol.

[15] Tolstrup M-B, Watt SK, Gögenur I (2017). Reduced rate of dehiscence after implementation of a standardized fascial closure technique in patients undergoing emergency laparotomy. Ann Surg.

[16] Ah-kee EY, Kallachil T, O’Dwyer PJ (2014). Patient awareness and symptoms from an incisional hernia. Int Surg.

[17] Bosanquet DC, Ansell J, Abdelrahman T, Cornish J, Harries R, Stimpson A, Davies L, Glasbey JCD, Frewer KA, Frewer NC, Russell D, Russell I, Torkington J (2015) Systematic review and meta-regression of factors affecting midline incisional hernia rates: analysis of 14 618 patients. PLoS One 10:. 10.1371/journal.pone.0138745 10.1371/journal.pone.0138745 PMC4577082 26389785

[18] Karlsson N, Zackrisson S, Buchwald P (2020) Computed tomography verified prevalence of incisional hernia 1 year postoperatively after colorectal cancer resection. Scand J Surg 1457496920976053. 10.1177/1457496920976053 10.1177/1457496920976053 PMC8551438 33326354

[19] Millbourn D (2009). Effect of stitch length on wound complications after closure of midline incisions: a randomized controlled trial. Arch Surg.

[20] Albertsmeier M, Hofmann A, Baumann P, Riedl S, Reisensohn C, Kewer JL, Hoelderle J, Shamiyeh A, Klugsberger B, Maier TD, Schumacher G, Köckerling F, Pession U, Weniger M, Fortelny RH (2022). Effects of the short-stitch technique for midline abdominal closure: short-term results from the randomised-controlled ESTOIH trial. Hernia.

[21] Walming S, Angenete E, Block M, Bock D, Gessler B, Haglind E (2017). Retrospective review of risk factors for surgical wound dehiscence and incisional hernia. BMC Surg.

[22] de Beaux AC (2019). Abdominal wall closure. Br J Surg.

[23] de Vries HS, Verhaak T, van Boxtel TH, van den Heuvel W, Teixeira MB, Heisterkamp J, Zimmerman DDE (2020). Implementation of the small bites closure of abdominal midline incisions in clinical practice is correlated with a reduction in surgical site infections. Hernia J Hernias Abdom Wall Surg.

[24] Söderbäck H, Gunnarsson U, Hellman P, Sandblom G (2018). Incisional hernia after surgery for colorectal cancer: a population-based register study. Int J Colorectal Dis.

[25] Söderbäck H, Gunnarsson U, Martling A, Hellman P, Sandblom G (2019). Incidence of wound dehiscence after colorectal cancer surgery: results from a national population-based register for colorectal cancer. Int J Colorectal Dis.

[26] Garcia-Urena MA, POP (Progress On Prevention) Surgical Group (2021). Preventing incisional ventral hernias: important for patients but ignored by surgical specialities? A critical review. Hernia J Hernias Abdom Wall Surg.

[27] Bloemen A, De Kleijn RJCMF, Van Steensel S, Aarts F, Schreinemacher MHF, Bouvy ND (2019). Laparotomy closure techniques: do surgeons follow the latest guidelines? Results of a questionnaire. Int J Surg Lond Engl.

