# Supplementary material for: Introduction of Small Stitch Small Bite technique: a retrospective long-term follow-up
Source: Langenbecks Arch Surg. 2022 May 17;407(6):2527–35. doi: 10.1007/s00423-022-02530-8 (PMC9467962; doi:10.1007/s00423-022-02530-8)
Supplement: Supplementary file 1 — Supplementary file1 (DOCX 82 KB) [file 423_2022_2530_MOESM1_ESM.docx]

Guidelines on opening and closure of abdominal incisions.

**Background***:*

Midline incisions are associated with severe complications such as wound dehiscence, wound infection, and incisional hernia. There is reliable evidence that the most effective way of reducing these complications is meticulous surgical technique.

Our department has decided, without exception, to use the so-called Sundsvall technique for closure of all midline abdominal incisions.

**Opening:**

- Spend time making your incision precisely in the midline. Apply the technique of subcutaneous fat fractioning if possible.
- Free the aponeurosis from subcutaneous tissue 1 cm on each side before it is incised, in preparation for closure.
- Use electrocautery to achieve clean aponeurosis edges for stitching.

**Closure**:

- Measure the incision length in the relaxed position. Use a ruler.
- Use PDS II 2-0, 150 cm, CT-1 needle.
- Suture length/wound length quote is measured by first measuring the incision length. The total length of the suture used minus the lengths cut off at the ends is assessed. Division of the suture length by the wound length gives the ratio.
- If the incision is longer than 30 cm you will need 2 sutures to reach a quote of at least 4.
- Use self-locking start and stop knots.
- Make sure to start at the very end of the incision.
- Start at the end of the incision and suture continuously using small bites 5-8mm from the aponeurosis edges. The interval between the stiches should be less than 5mm.
- Take bites in the aponeurosis only. Avoid mass layered sutures. If you leave the midline, take bites in the anterior rectus aponeurosis only.
- A low-tension suture is important, pull the suture so that the edges of the aponeurosis just adapt. The assistant should hold the suture without increasing the tension. The suture must be visible but the distance between the edges should be <10mm
- Suture length/wound length must be more than 4, and the ratio must be measured and noted in the operation notes.
- If a ratio of four is not achieved, the suture must be redone. The ratio has no maximum.
- The theatre nurse has the responsibility to reach the surgeon a pair of scissors if a ratio of 4 is not achieved. If the surgeon choses to deviate from this routine, a non-compliance report must be made stating the reasons for this.
- The skin must be closed with intracutaneous Monocryl^®^ suture. If this is not possible, staples may be used.

**References**:

Effect of stitch length on wound complications after closure of midline incisions: a randomized controlled trial.

Millbourn D, Cengiz Y, Israelsson LA. Arch Surg. 2009 Nov;144(11):1056-9

Mass closure technique: an experimental study on separation of wound edge. Cengiz Y, Gislason H, Svanes

K, Israelsson LA. Eur J Surg. 2001 Jan;167(1):60-3.

Risk factors for wound complications in midline abdominal incisions related to the size of stitches. Millbourn D,

Cengiz Y, Israelsson LA. Hernia. 2011 Jun;15(3):261-6. Epub 2011 Jan 30.

Small tissue bites and wound strength: an experimental study. Cengiz Y, Blomquist P, Israelsson LA. Arch

Surg. 2001 Mar;136(3):272-5.

The surgeon as a risk factor for complications of midline incisions. Israelsson LA. Eur J Surg. 1998

May;164(5):353-9
